# Implementing a “free” tuberculosis (TB) care policy under the integrated model in Jiangsu, China: practices and costs in the real world

**DOI:** 10.1186/s40249-016-0099-8

**Published:** 2016-01-22

**Authors:** Xinxin Jia, Jiaying Chen, Siyuan Zhang, Bing Dai, Qian Long, Shenglan Tang

**Affiliations:** Center for Health Policy Studies, Nanjing Medical University, Hanzhong Road 140, 210029 Nanjing, China; The Second Affiliated Hospital of Soochow University, Suzhou, Jiangsu Province China; Zhenjiang Center for Disease Control and Prevention, Zhenjiang, Jiangsu Province China; Duke Global Health Institute, Duke University, Durham, NC USA; Global Health Research Center, Duke Kunshan University, Kunshan, China

**Keywords:** Tuberculosis, Anti-TB treatment practice guidelines, Free, Expenditure, Jiangsu province, China

## Abstract

**Background:**

In the 1990s, China introduced a “free” tuberculosis (TB) care policy under the national TB control program. Recently, as a part of a new TB diagnosis and treatment model, it has been recommended that the integrated model scale up. This paper examines whether or not TB designated hospitals in the selected project sites have provided TB care according to the national and local guidelines, and analyzes the actual practices and expenditures involved in completing TB treatment. It also explores the reasons why “free” TB care in China cannot be effectively implemented under the integrated model.

**Methods:**

This study was conducted in three counties of Zhenjiang city, Jiangsu province. Mixed methods were used, which comprised reviewing the national and local TB control guidelines, conducting TB patient surveys, collecting TB inpatient and outpatient hospital records, and conducting qualitative interviews with stakeholders. Descriptive statistics were used for quantitative data analysis across counties and in order to compare patients who received only outpatient care and those who received both outpatient and inpatient care. The chi-square test and analysis of variance were performed where necessary. Qualitative data were analyzed using the framework approach.

**Results:**

Although the national TB care guidelines recommend outpatient care as a basis for TB treatment in China, we found high hospital admission rates for TB patients ranging from 39 % in Yangzhong county to 83 % in Dantu county. Almost all outpatient TB patients paid for lab tests and over 80 % paid for liver protection drugs and around 70 % paid for image examinations. These three components accounted for three-quarters of the total outpatient expenditure. For patients who received only outpatient care, the total expenditure upon completion of TB treatment was on average 1,135 Chinese yuan. For patients who received outpatient and inpatient care, the total expenditure upon completion of TB treatment was 11,117 Chinese yuan.

**Conclusion:**

The “free” TB care policy under the integrated model has not been effectively implemented in China. There has been substantial spending on non-recommended services, examinations, and drugs for TB treatment.

**Electronic supplementary material:**

The online version of this article (doi:10.1186/s40249-016-0099-8) contains supplementary material, which is available to authorized users.

## Multilingual abstracts

Please see Additional file 1 for translations of the abstract into the six official working languages of the United Nations.

## Background

Tuberculosis (TB) remains a serious public health issue worldwide. China has the world’s second largest TB epidemic with an estimate of one million new cases reported in 2013, which accounted for 11.6 % of the global incidence [1].

Fighting against TB has gained high political commitment in China. In the 1990s, China initiated an innovative national TB control program (NTP) with the World Health Organization (WHO)-recommended directly observed treatment, short-course (DOTS) strategy funded by a World Bank loan and the Chinese Ministry of Health (now called the National Health and Family Planning Commission of China, NHFPC) [2, 3]. To remove financial barriers to access to standard TB diagnosis and treatment, especially for the poor, healthcare providers in general health facilities are required to refer TB suspects to local TB dispensaries for diagnosis based on smear microscopy and radiography free of charge. Patients diagnosed with smear-positive or severe smear-negative TB could be treated with free first-line anti-TB drugs at TB dispensaries [4]. This policy has gradually been extended to cover all smear-negative TB patients, and was expanded to the entire country by 2005 [3] as part of the “free” TB diagnosis and treatment policy.

From 1990 to 2010, China has more than halved its TB prevalence, which is largely attributed to the DOTS program and the free TB treatment policy [2]. The WHO called China’s NTP-DOTS program “one of the most successful DOTS programs in the world.” But although TB prevalence has dropped significantly, there is still a heavy TB burden in China. The 2010 national TB epidemiology survey reported that the prevalence of active pulmonary TB was 459 per 100,000 population, and the prevalence of TB in rural areas was almost twice that of urban areas [5]. What’s more, the convergence of TB management systems—which authorize only TB dispensaries and/or TB centers to provide TB diagnosis, treatment, and case management—have faced challenges regarding the treatment of multidrug-resistant TB and other TB-related complications [6]. In the late 1990s, a new model called the “integrated model” was piloted and carried out in some eastern provinces (Shanghai, Zhejiang, and Jiangsu) and a few sites in western China [7]. It encompassed the setup of TB clinics in general hospitals to provide standard TB care and case management in cooperation with local TB dispensaries. All TB patients are diagnosed and treated in designated hospitals and the local TB dispensaries are mainly in charge of TB public health care including health education, training and supervision. It is now recommended that this model scales up.

Despite gains in TB control, evidence has shown that there are substantial costs associated with TB diagnosis and treatment, and that TB patients face a heavy financial burden, even under the “free” TB care policy [8–11]. Many previous studies found that TB patients were charged for longer treatment periods than recommended, and drugs and tests were administered beyond the standard treatment regimen, all largely attributed to perverse financial incentives of TB care providers [12]. Although TB diagnosis and treatment has shifted to TB designated hospitals, little is known about the implementation of the national TB care policy and the costs of TB care in these hospitals.

The main objectives of this paper are to examine whether or not TB designated hospitals in the selected project sites have provided TB care according to the national and local practice guidelines, and to determine the actual practices and costs involved in completing TB treatment. The paper also explores the reasons why “free” TB care in China cannot be effectively implemented under the integrated model.

## Methods

### Study design

For the purpose of this study, we used data collected from Zhenjiang city, which is a prefectural city consisting of several urban districts and county-level cities (we refer to these as “counties” in this paper), in Jiangsu province. Zhenjiang was selected as it is one of the project cities of the China NHFPC and the Gates Foundation TB Project (China-Gates TB project for short) Phase II. In 2002, the integrated model was initiated in counties in Zhenjiang. All county general hospitals were authorized to act as TB designated hospitals; that is, they were in charge of the diagnosis and treatment of TB patients who lived in the respective counties. Data collection was carried out in Jurong (JR), Dantu (DT), and Yangzhong (YZ) counties, which were selected according to their gross domestic product per capita in 2012, and classified as low, middle and high income, respectively (54,140 yuan in JR, 83,388 yuan in DT, 105,879 yuan in YZ). The integrated model was implemented in DT county in 2002, and in the YR and JR counties in 2011.

### Data collection

A mixed method was used to collect the data. Both quantitative and qualitative methods were incorporated to complement each other, making the study more comprehensive.

Firstly, we collected the national and local TB diagnosis and treatment practice guidelines from the TB designated hospitals that were focused on the free TB care policy to examine whether TB care providers adhered to the guidelines.

Secondly, a patient survey was carried out in the three selected counties to investigate the use of and expenditures associated with TB care. A cluster random sampling method was adopted based on townships and streets to recruit TB cases who started a TB treatment course in 2012 and completed or stopped treatment before the commencement of the survey (April 2013). In each county, three townships/streets were randomly selected and, in each of those, 30 TB cases were randomly sampled from the TB case registration list. We sampled a few TB cases from 2011 when the TB cases from 2012 didn’t fulfill the sample size requirement. In total, 267 TB patients were identified and interviewed using a structured questionnaire by trained medical students from Nanjing Medical University. The questionnaires collected information on: patient demographic and social factors, TB-related diagnostic and treatment pathways/histories, direct health service expenditures and indirect expenses (e.g. transportation and accommodation costs, lost household income, etc.), and reimbursements from health insurance agencies. After data cleaning, there were 263 valid, completed questionnaires.

Thirdly, complete medical records of the sampled patients were obtained from their respective county’s designated hospital. We used patients’ names as a key variable to find outpatient records of patients who completed a full course of TB treatment. Tuberculosis inpatient records from 2010 to 2012 were also collected. Both inpatient and outpatient records included information on frequency of care use, services used (e.g. laboratory and X-ray examinations, and drug regimens and prescriptions), and itemized expenditures. Fees for services covered by the free TB care policy were directly deducted and not recorded in the patients’ medical records.

In addition, qualitative interviews were conducted with stakeholders in the selected three counties to explore their perceptions about adherence to TB practice guidelines, especially how it relates to the implementation of the free TB care policy in the designated hospitals. These consisted of key informant interviews (KII) and focus group discussions (FGDs). Semi-structured KIIs were conducted with local health administrators, Center for Disease Control and Prevention (CDC) heads who were in charge of TB control, CDC TB control unit leaders, heads of TB designated hospitals, and local health insurance managers; 15 individual interviews were conducted. The KIIs wanted to elucidate information on the financial burden of TB patients, health insurance regulations on service provision, and payment methods for TB treatment. The FGDs were carried out with TB care providers in the county designated hospitals; three FGDs were conducted. Each focus group had eight to ten participants, comprising doctors (two to three) and nurses (two to three) who treat TB, the head of the TB unit, lab staff (one to two), and administrative staff (one to two) who worked in TB diagnosis and treatment. The FGDs wanted to elucidate information on patient treatment adherence, doctor/nurse incomes and bonuses compared to their counterparts in other departments, career development opportunities, job perception and satisfaction, TB diagnosis and treatment practices, and how participants perceived the provision of TB diagnosis and treatment.

### Data analysis

We reviewed the national and local TB care practice guidelines and summarized information pertaining to DOTS implementation, services used (e.g. laboratory and X-ray examinations, and drug regimens and prescriptions), and the free TB care policy.

In terms of quantitative data, we linked the patient surveys (263 cases) with the patients’ respective hospital records in order to acquire precise information on the use of and expenditure relating to full-course TB treatment. We successfully matched 200 cases (hereinafter referred to as a “linked dataset”). The four indicators we examined were: 1) hospital admission rate, calculated by the number of admitted TB patients as a proportion of the total number of TB patients undertaking the survey; 2) frequency of outpatient visits based on the linked dataset; 3) proportion of patients paying for lab tests (including blood, urine, liver function, or kidney function tests, etc.), and image examinations (including chest radiography, X-ray examination, and CT); and 4) proportion of patients paying for non-free anti-TB drugs and liver protection drugs according to the linked dataset. The total medical expenditures upon completing TB treatment were determined based on the hospital records in the linked dataset. Descriptive statistics were used to examine TB care use and expenditures associated with TB care among patients who received only outpatient care and those who received both outpatient and inpatient care. Itemized expenditures relating to outpatient care among patients who received only outpatient care were also examined.

Data collected using qualitative methods were recorded and transcribed, and then analyzed using the framework approach. The framework was developed based on the topic guide and categories that emerged from the transcripts, and was used to identify themes. All qualitative data were coded, sorted, and classified in terms of this framework. Charting was used to identify common and divergent perceptions, and explanations were developed. The NVivo 10 software package was used to manage data.

### Ethical considerations

The study design and implementation of the China-Gates TB project Phase II was approved by the Research Ethics Committee of the China CDC. All data were collected with the informed consent of participants prior to their participation in the study. Data access complied with standard procedures.

## Results

### National and local TB care practice guidelines

The national TB care practice guidelines [4] recommend that TB treatment predominantly consists of outpatient care for six to eight months. Tuberculosis patients are urged to visit TB designated facilities once a month to take anti-TB drugs and undergo recommended tests and examinations. Specifically, it is recommended that sputum smear tests are conducted by the end of the second, fifth, and sixth months of treatment (for new patients), or the eighth month of treatment (for relapsed patients). In addition, routine blood, urine, and liver function tests are recommended at treatment initiation. By the end of the first month of treatment, routine blood and urine tests are also recommended. Liver function tests can be performed if necessary (e.g. if side effects are reported by patients in relation to abnormal liver function). X-ray examination is recommended at treatment initiation, by the end of the first month of treatment, and at the end of the treatment. During treatment, it’s only necessary for TB patients with severe complications to be admitted to hospital. Across the full course of treatment, two X-ray examinations, three sputum smear tests, and first-line anti-TB drugs are provided free of charge.

In addition to the national “free” TB policy, two liver function tests and one or two chest radiographies are free of charge for TB patients in Zhenjiang. Fixed-dose combinations (FDCs) of the first-line anti-TB drugs are commonly used. In DT county, township doctors collect anti-TB drugs from the TB designated hospital every two months, allowing TB patients to take their drugs and conduct their tests in township health centers.

### Comparison between the patient surveys (263 cases) and the linked dataset (200 cases)

Two hundred and sixty-three TB patients participated in the patient survey, however, only 200 complete medical records were successfully obtained from hospitals. Out of the 200 patients with complete medical records, 106 received only outpatient care and 94 received both outpatient and inpatient care. After comparing the two datasets, no significant differences were found in respect to age, gender, type of residence, type of health insurance, or hospital admission rate (*p* = 0.870, *p* = 0.999, *p* = 0.649, *p* = 0.435, and *p =* 0.116, respectively). This means that how these 200 patients use TB care and the costs associated with completing treatment can represent the entire cohort of 263 patients. Therefore, we used a linked dataset (200 cases) to examine outpatient care utilization and precise expenditure on completing TB treatment.

### Characteristics of TB patients

Table 1 presents the demographic and social characteristics of the surveyed TB patients. Of the 263 patients, 193 were new patients and 70 were relapsed patients. The majority of TB patients were rural residents (89.4 %) and male (73 %), and more than half were over 60 years of age (57.4 %). Almost all patients had health insurance coverage; 82 % were covered by the New Cooperative Medical Scheme (NCMS), designed for the rural population and oriented at inpatient services, implemented by the central Chinese government. Similar results were found across the three counties.

**Table 1:** Demographic and social characteristics of TB patients by county %, 2012

	DT (*n* = 84)	YZ (*n* = 82)	JR (*n* = 97)	Total (*n* = 263)
Age				
<30	3.6	7.3	6.2	5.7
30–59	45.2	32.9	33.0	36.9
60+	51.2	59.8	60.8	57.4
Sex				
Male	69.0	74.4	75.3	73.0
Female	31.0	25.6	24.7	27.0
Type of residence				
Rural	96.4	84.2	87.6	89.3
Urban	3.6	14.6	11.4	9.9
Unified residence (*hukou*)^b^	0.0	1.2	1.0	0.8
Health insurance				
None	0.0	6.1	0.0	1.9
NCMS	89.3	68.3	86.6	81.7
Other^a^	10.7	25.6	13.4	16.4

Data source: TB patient survey

^a^Other included the Urban Employee Basic Medical insurance, the Urban Residence Basic Medical insurance, and commercial health insurance

^b^ *hukou* is the household registration system in China. It’s usually a dichotomy—rural *hukou* or urban *hukou*. In rare cases, it may be unified

### Utilization of TB care

#### Inpatient care

The patient survey revealed that half of the participating TB patients had been admitted to hospital. The proportion of hospital admission was highest in DT county (83 %), followed by JR county (43 %) and YZ county (39 %). The difference between the respective counties was statistically significant (*χ*^2^ = 41.021, *p* < 0.0001). The length of hospital stay was also longest in DT county (33.4 days), compared to both JR and YZ counties (22 days). No statistically significant difference was observed in the hospital admission rate between new TB patients and relapsed patients (*χ*^2^ = 0.870, *p* = 0.401).

Local CDC staff and healthcare providers in county TB designated hospitals had different opinions regarding the high hospital admission rates. In all three counties, almost all CDC heads in charge of TB control and TB unit leaders thought that overprovisioning of services, driven by financial incentives to TB designated hospitals, resulted in the high hospital admission rates and in turn increased the financial burden placed on TB patients. In contrast, some healthcare providers in TB designated hospitals thought smear-positive TB patients should be admitted to hospital to reduce and/or avoid community infection. Some explained that some TB patients asked to be admitted, as they could then claim reimbursement for hospital admission but could not claim for outpatient care. In addition, some healthcare providers believed that high hospital admission rates were partly attributed to fear of malpractice and conflict with patients.

#### Outpatient care

According to the linked dataset, patients who only received outpatient care on average visited the outpatient department 7.5 times—more than those who received both inpatient and outpatient care (see Table 2). The frequency of outpatient visits was lowest in DT county, followed by YZ county, and was the highest in JR county. There was no statistically significant difference observed in outpatient visits between new and relapsed patients in all three counties (*p* = 0.889 in DT, *p* = 0.201 in YZ, *p* = 0.222 in JR). The high admission rate in DT (83 %) and the practice where township doctors collect anti-TB drugs from the county TB designated hospital every two months and dispense them to patients is probably why so few patients seek outpatient care in DT county.

**Table 2:** Frequency of outpatient visits during a treatment course among patients who received only outpatient care and patients who received both outpatient and inpatient care by county %, 2012

	DT	YZ	JR	Total
Receiving only outpatient care	(*n* = 14)	(*n* = 49)	(*n* = 43)	(*n* = 106)
1–3	78.6	0.0	20.9	18.9
4–6	7.1	36.7	14.0	23.6
7–9	14.3	51.0	16.3	32.1
10+	0.0	12.3	48.8	25.4
(Mean)	(2.9)	(7.3)	(9.3)	(7.5)
Receiving both outpatient and inpatient care	(*n* = 61)	(*n* = 10)	(*n* = 23)	(*n* = 94)
1–3	83.6	10.0	8.7	57.4
4–6	8.2	60.0	17.4	16.0
7–9	1.6	20.0	17.4	7.4
10+	6.6	10.0	56.5	19.2
(Mean)	(3.1)	(6.3)	(11.4)	(5.5)

Data source: Patient surveys and hospital records

In addition to the services covered by the national and local free TB policies, almost all TB patients paid for lab tests (including the practice guideline-recommended and non-recommended tests), and a vast majority paid for liver protection drugs in all three counties (see Table 3). In addition, around 70 % of patients paid for image examinations (e.g. CT). Of the patients who received only outpatient care, nearly 40 % took non-free, second-line anti-TB drugs, whereas the proportion of patients taking non-free anti-TB drugs was much lower for those who received both inpatient and outpatient care (19.1 %).

**Table 3:** Proportion of patients paying for drugs, lab tests, and examinations during outpatient visits by county (%), 2012

	DT	YZ	JR	Total
Receiving only outpatient care	(*n* = 14)	(*n* = 49)	(*n* = 43)	(*n* = 106)
- Non-free anti-TB drugs	42.9	36.7	41.9	39.6
- Liver protection drugs	64.3	85.7	83.7	82.1
- Image examination^a^	85.7	77.6	53.5	68.9
- Lab tests^b^	100.0	98.0	100.0	99.1
Receiving outpatient and inpatient care	(*n* = 61)	(*n* = 10)	(*n* = 23)	(*n* = 94)
- Non-free anti-TB drugs	18.0	10.0	26.1	19.1
- Liver protection drugs	75.4	90.0	91.3	80.9
- Image examination^a^	82.0	80.0	43.5	72.3
- Lab tests^b^	95.1	100.0	100.0	96.8

Data source: Patient surveys and hospital records

^a^Image examination included X-ray examination, chest radiography, and CT

^b^Lab tests included blood test, urine test, liver function test, kidney function test, etc

Qualitative interviews explored perceptions of stakeholders about outpatient care provision with a focus on the free TB care policy. In the three counties, almost all CDC staff, TB designated hospital managers, and healthcare providers expressed that only a few services were covered by the free TB care policy, and “many necessary tests and drugs, such as liver function tests and liver protection drugs, among others, were not free and expensive.” Some CDC TB unit leaders mentioned that “[doctors of] TB designated hospitals often performed CT instead of X-ray examinations and prescribed non-free anti-TB drugs,” which was perceived to be unnecessary and costly. Most healthcare providers in the TB designated hospitals said that their patients suffered from side effects and this was the main reason for prescribing non-free anti-TB drugs. Others added that it was difficult to address patients’ side effects caused by one or two anti-TB drugs when FDC drugs were used. In addition, several healthcare providers in the designated hospitals expressed mistrust in the effectiveness and quality of the free anti-TB drugs.

### Expenditure associated with TB treatment

We analyzed expenditure associated with completing TB treatment among patients who only received TB outpatient care and those who received both outpatient and inpatient care, using the hospital records in the linked dataset. For patients who received only outpatient care, the total expenditure upon completion of TB treatment was on average 1,135 Chinese yuan and approximately 151 Chinese yuan per visit. Costs associated with lab tests, image examinations, and liver protection drugs accounted for three-quarters of the total expenditure. Expenditure for outpatient TB treatment was highest in JR county, followed by YZ county, and was lowest in DT county (see Table 4). In terms of itemized expenditures, image examinations in DT and YZ counties and liver protection drugs in JR county accounted for around one-third of the total expenditure. For patients who received both outpatient and inpatient care, total expenditure upon completion of treatment was almost 10 times that of expenditure associated with receiving only outpatient care (see Table 5). Overall, the expenditure for both outpatient and inpatient care in YZ county was relatively lower than in DT and JR counties.

**Table 4:** Medical expenditure on TB treatment (in Chinese yuan) incurred by patients receiving only outpatient care, by county 2012

	DT (*n* = 14)	YZ (*n* = 49)	JR (*n* = 93)	Total (*n* = 106)
Expenditure per outpatient visit	233.8	155.9	137.4	150.6
Total expenditure per patient^a^	684.7	1,139.3	1,277.7	1,135.4
- Lab tests	266.6	253.7	361.8	299.3
(as a % of the total expenditure)	38.9 %	22.3 %	28.3 %	26.4 %
- Image examination	256.2	395.7	51.9	237.8
(as a % of the total expenditure)	37.4 %	34.7 %	4.1 %	20.9 %
- Non-free TB drugs	20.7	95.5	47.9	66.3
(as a % of the total expenditure)	3.0 %	8.4 %	3.8 %	5.8 %
- Liver protection drugs	87.8	288.2	452.3	328.3
(as a % of the total expenditure)	12.8 %	25.3 %	35.4 %	28.9 %
- Other	53.4	106.2	363.8	203.8
(as a % of the total expenditure)	7.8 %	9.3 %	28.5 %	18.0 %

Data source: Patient surveys and hospital records

^a^ANOVA for total expenditure per patient between the three counties, *p* < 0.0001, significant difference observed between DT county and JR county

**Table 5:** Medical expenditure on TB treatment (in Chinese yuan) incurred by patients receiving both outpatient and inpatient care, by county 2012

	DT (*n* = 61)	YZ (*n* = 10)	JR (*n* = 23)	Total (*n* = 94)
Outpatient care expenditure				
Expenditure per visit	209.4	195.2	167.7	186.3
Total outpatient expenditure	645.3	1,229.6	1,902.8	1,015.1
Inpatient care expenditure				
Expenditure per day	327.1	344.4	415.3	344.6
Total inpatient expenditure	10,921.3	7369.1	9,117.6	10,102.1
Total^a^	11,566.6	8,598.7	11,020.4	11,117.2

Data source: Patient surveys and hospital records

^a^ANOVA for total cost between three counties, *p* = 0.598, no significant difference observed in the total expenditure for patients receiving both outpatient and inpatient care between counties

Qualitative interviews with health insurance managers elucidated that most outpatient TB care services, including lab tests, CT scans, and liver protection drugs, were not covered by the NCMS. Tuberculosis patients paid for outpatient care fully out-of-pocket in YZ, and largely in DT and JR. Although health insurance managers in the three counties said that health insurance schemes usually covered 70–80 % of inpatient expenditure, aiming to reduce the patients’ financial burden, some CDC leaders indicated that many prescribed services and drugs were not included in the benefit packages of the health insurance schemes. Hence, the actual reimbursement proportion was much lower, suggesting that patients faced a heavy financial burden due to TB treatment.

## Discussion

Our study showed that China’s NTP provides TB patients with “free” TB care for essential diagnosis and treatment based on the national policies developed by the central government.

However, the extent to which free TB care is being provided varies from place to place, subject to local resources and policies. This study revealed that the three project counties in Jiangsu province offered more free lab tests and chest radiographies than other counties in China.

In the context of free essential TB care and near universal health coverage, TB patients should not experience much financial difficulty in accessing TB care. Unfortunately, this study found that this is not the reality for those seeking treatment under the integrated model. First, the policies of the NCMS do not offer adequate coverage of outpatient services, including TB outpatient care. For one reason or another, the national guidelines for developing NCMS policies, issued by the central government in the early 2000s, requested local authorities at county and city levels to prioritize coverage of inpatient services. However, a vast majority of TB patients may only need TB diagnoses and treatment, which can be provided at hospitals’ outpatient departments. Furthermore, Chinese hospitals often provide TB patients with extra services and medicines, such as liver protection drugs, non-free, second-line anti-TB drugs, and extra tests, which are not on the reimbursable list approved by the NCMS, nor on the list of free TB services. In other words, and as we found in this study, most TB outpatient service expenses were paid for out-of-pocket by TB patients. Many studies published in China and elsewhere support these findings [13, 14].

Expenditure associated with inpatient services is much higher than that associated with outpatient services. Tuberculosis care is no exception. Many papers have reported that Chinese hospitals often maximized revenue generation through the overprovision of healthcare services [15–17]. Hence, it is not surprising that the rates of hospital admission for TB patients in the three project sites ranged from 39 % in YZ county to 83 % in DT county. Hospitalized TB patients often believe that the NCMS policy partially covers expenses associated with inpatient services. What they likely did not realize was that the amount required to pay out-of-pocket, as part of the deductible and co-insurance payments, would be much higher than what they were otherwise required to pay for outpatient services. Our study shows that the expenditure for only outpatient care was just one-tenth of what it was for both outpatient and inpatient care, which is consistent with other studies conducted in both developed and developing countries [18, 19]. Hospital admission for patients with uncomplicated TB is not necessary and should be mitigated or avoided in order to use resources in a more cost-effective manner. This is particularly important in settings with limited health resources.

One thing is clear—the hospitals included in this study did not follow the national guidelines for TB care and control in China. They intended to overprovide TB services, including overusing second-line anti-TB drugs. However, they may have had their reasons for this. For example, healthcare providers at hospitals might have wanted patients with complications or those experiencing side effects related to the use of first-line drugs to be admitted to hospital, and therefore also administered second-line anti-TB drugs. However, many published studies [8–10] and our qualitative findings indicate that although these might be justifiable reasons for higher hospital admission rates and/or for the overuse of second-line anti-TB drugs or examinations, the unacceptable financial incentives given to hospitals in China may be one of the main factors resulting in this overprovisioning of TB services. In the 1980s, with the shrinking of government investment in public health facilities, public hospitals gained increasing financial autonomy to generate revenues and retain surpluses. Meanwhile, salaries of healthcare providers were tied with revenue generated for facilities through a bonus system. Since then, free standing public hospitals have competed for patients by introducing highly technological medical services, providing comprehensive diagnostic and laboratory tests, and encouraging patients to take expensive new drugs, known as the “medical arms race” [20, 21]. Driven by profit, healthcare providers opted to provide more and more expensive services.

The overprovisioning of TB care has many implications. The two chief ones are 1) increased financial burdens placed on TB patients’ families and health insurance funds, and 2) compromising the quality of care that may lead to more drug-resistant patients. Inappropriate and/or interrupted treatments have been identified as the main causes for drug-resistant TB development in China, and are associated with adverse health outcomes [22, 23]. Our study and other studies in China [8–10] found that substantial expenditures were incurred by TB patients and were the result of many non-recommended services, which have culminated in discontinued treatment due to difficulty in affording care [24]. Several papers included in this special issue paint a grave picture of the catastrophic expenditures incurred by TB patients who have completed treatment due largely to high out-of-pocket payments. Patients are at great risk of falling into the vicious disease-poverty cycle. Moreover, unnecessary medical services will increase consumption of the pool fund of health insurance schemes and may take limited resources away from necessary medical service coverage. The overprovisioning of TB care not only undermines efforts to control TB, but also results in serious socioeconomic consequences for poor TB patients.

## Conclusion

It appears that the free TB care policy cannot effectively cover the requirements of diagnosis and treatment for TB under the integrated model. Both the “free” TB care policy and the NCMS benefit package do not work under this model to successfully control TB in Jiangsu, China, as we have illustrated in this paper. The Chinese government needs to take action to regulate and improve the rational provisioning of TB care by hospitals via the development of a sensible mechanism for financing hospitals. It must also improve the benefit packages offered by the NCMS, so these can provide more coverage for outpatient care. Inaction on these issues could bring about further grave consequences for TB care and control in the coming years.

### Study strengths and limitations

In this study, we examined the use of and expenditures associated with TB care based on hospital records, which is a more accurate method than patient recall. Moreover, combined quantitative and qualitative data provide holistic explanations for TB care provision and its implications. However, there are some limitations. Firstly, the hospital records reflected total medical expenditure for TB treatment, but were not stratified to show how much of the expenditure was reimbursable by health insurance schemes or how much patients paid out-of-pocket. In addition, we couldn’t identify 63 of the 263 surveyed TB patients using the hospital records primarily due to incomplete information. The sample size was relatively small and generalization of results should be done with caution.

## Additional file

Additional file 1:
**Multilingual abstracts in the six official working languages of the United Nations.** (PDF 295 kb)

## Abbreviations

CDC: Center for Disease Control and Prevention
CT: Computer tomography
DOTS: directly observed treatment, short-course
DT: Dantu county
FDC: fixed-dose combination
FGD: focus group discussion
JR: Jurong county
KII: key informant interview
NCMS: New Cooperative Medical Scheme
NHFPC: National Health and Family Planning Commission
NTP: national tuberculosis control program
TB: Tuberculosis
WHO: World Health Organization
YZ: Yangzhong county
