# Supplementary material for: Implementing a “free” tuberculosis (TB) care policy under the integrated model in Jiangsu, China: practices and costs in the real world
Source: Infect Dis Poverty. 2016 Jan 22;5:1. doi: 10.1186/s40249-016-0099-8 (PMC4722763; doi:10.1186/s40249-016-0099-8)
Supplement: Additional file 1: — Multilingual abstracts in the six official working languages of the United Nations. (PDF 295 kb) [file 40249_2016_99_MOESM1_ESM.pdf]

Translation of the abstract into the six official working languages of the United Nations

تنفيذ سياسة الرعاية "المجانية" من مرض السل بموجب النموذج المتكامل في جيانجسو، الصين، الممارسة والتكلفة في العالم الحقيقي.

زين زين [1]، جيانج تشن[1]*، سيان تزانج[2]، بينج داي[3]، كيان لونج[4،5]، وشينجلان تانج[4،5]

ملخص

الخلفية: في تسعينات القرن العشرين، قدمت الصين سياسة رعاية "مجانية" ضد مرض السل بموجب البرنامج الوطني لمكافحة مرض السل. مؤخرًا، وكجزء من التشخيص الجديد لمرض السل ونموذج معالجته، تمت التوصية على يتم زيادة معدل النموذج المدمج. تقوم هذه الورقة بدراسة ما إذا كانت مستشفيات تشخيص مرض السل في مواقع المشروع المختارة قدمت رعاية مرض السل وفقًا للإرشادات الوطنية والمحلية وقامت بتحليل الممارسات الفعلية والمصروفات الخاصة باستكمال علاج مرض السل أم لا. كما أنها تبحث في الأسباب وراء عدم إمكانية تنفيذ الرعاية المجانية ضد مرض السل في الصين بفعالية بموجب النموذج المدمج.

الطرق: لقد تم إجراء تلك الدراسة في ثلاث مقاطعات من مدينة زينجيان، في ولاية جيانسو. تم استخدام طرق مختلطة، والتي تتكون من مراجعة الإرشادات الوطنية والمحلية لمكافحة مرض السل وإجراء دراسة على مرضى السل وجمع سجلات المستشفيات لمرض العيادات الخارجية والمرضى المقيمين في المستشفى وإجراء مقابلات نوعية مع المعنيين. تم استخدام إحصاءات وصفية لتحليل البيانات النوعية عبر المقاطعات ومن أجل مقارنة المرضى الذين تلقوا رعاية في العيادات الخارجية فقط ومن تلقوا الرعاية كمرضى العيادات الخارجية ومرضى مقيمين في المستشفى. تم إجراء اختبار مربع كاي وتحليل المتغير متى كان ذلك ضروريًا. تم تحليل البيانات النوعية باستخدام مقاربة إطار العمل.

النتائج: برغم أن الإرشادات الوطنية الخاصة بالرعاية ضد مرض السل توصي بتقديم الرعاية للمرضى الخارجين كأساس لعلاج مرض السل في الصين، فإننا وجدنا معدلات تسجيل دخول للمستشفيات مرتفعة بالنسبة لمرضى السل تتراوح من 39% في مقاطعة يانجهونج إلى 83% في مقاطعة دانتو. تقريبًا كافة مرضى السل الخارجيين قاموا بسداد تكلفة اختبارات المعامل وأكثر من 80% منهم قام بسداد تكاليف أدوية حماية الكبد وحوالي 70% منهم سددوا تكلفة فحوصات الأشعة. تساوي تلك المكونات الثلاث ثلاثة أرباع إجمالي تكلفة المرضى الخارجيين. وبالنسبة للمرضى الذين يتلقون فقط الرعاية الخارجية، كان إجمالي المصروفات عند استكمال علاج مرض السل كان في المتوسط 1.135 يوان صيني. بالنسبة للمرضى الذين يتلقون رعاية كمرضى خارجيين ومن داخل المستشفى، كان إجمالي المصروفات حتى استكمال علاج مرض السل 11.117 يوان صيني.

الاستنتاج: لم يتم تنفيذ سياسة علاج مرض السل "المجانية" بموجب نموذج مدمج بصورة فعالة في الصين. لقد كان هناك إنفاق جوهري على الخدمات والفحوصات والأدوية الخاصة معالجة مرض السل غير موصى بها.

Translated from English version into Arabic by REHAM HUSSIEN, through

TRANSLATORS
WITHOUT BORDERS

“整合服务”模式下结核病“免费”治疗，以中国江苏为例---结核病诊疗行为及费用

贾欣欣，陈家应，张思源，戴冰，龙倩，汤胜蓝

摘要

目的：了解“整合服务”模式下，定点医院结核病诊疗行为及全疗程费用。

方法：采取分层随机抽样方法，抽取镇江 3 个县（区）普通结核患者为调查对象，采取定量和定性相结合的方法。调查包括结核患者问卷调查，政策文件收集与分析，定点医院门诊和住院病例收集和利益相关者访谈。定量资料的主要分析方法包括一般描述性分析和统计学检验，定性数据运用框架分析法。
结果：研究发现定点医院存在过度医疗的行为。尽管《结核病防治规划指南》规定普通结核患者以门诊治疗为主，本研究发现三家定点医院均存在较高的住院率，其中扬中 39%，丹徒 83%。国家结核病免费药物和检查项目以外，几乎所有的患者在门诊服务过程中都产生了化验费用，超过 80%的病人和约 70%的病人分别在门诊过程中产生了保肝药费用和 X-线检查费用，这三部分总费用占门诊总费用 3/4 以上。只门诊治疗的患者全疗程的总费用为 1135 元，既门诊又住院的患者全疗程总费用高达 11,117 元。
结论：“定点医院”模式下结核病免费治疗并未有效实施，结核病患者仍需支付较高的治疗费用。

Translated from English version into Chinese by Jia Xinxin

Mise en place d’une politique de soins « gratuits » contre la tuberculose suivant le modèle intégré au Jiangsu (Chine) : pratiques et coûts réels

Xinxin Jia[1], Jiaying Chen[1*], Siyuan Zhang[2], Bing Dai[3], Qian Long[4,5] et Shenglan Tang[4,5]

Résumé

Contexte : Dans les années 1990, la Chine a mis en place une politique de soins « gratuits » contre la tuberculose, dans le cadre du plan national contre la tuberculose. Une extension du modèle intégré a récemment été recommandée, suivant un nouveau modèle de diagnostic et de traitement de la maladie. Le présent article examine si les hôpitaux désignés pour la lutte contre la tuberculose sur les sites de projet sélectionnés ont ou non assuré les soins suivant les directives nationales et locales ; il analyse les pratiques locales et le coût pour mener à bien le traitement de la tuberculose. Il recherche aussi les raisons pour lesquelles les soins « gratuits » contre la tuberculose ne sont pas réalisables efficacement dans le modèle intégré.
Méthodes : L’étude a été menée dans trois circonscriptions de la ville de Zhenjiang, dans la province du Jiangsu. Plusieurs méthodes ont été combinées : revue des directives nationales et locales de lutte contre la tuberculose, enquêtes auprès de patients tuberculeux, consultation des dossiers d’hospitalisation et de consultations externes de ces patients, entretiens qualitatifs avec les parties prenantes. Des statistiques descriptives ont été utilisées pour quantifier l’analyse des données entre les circonscriptions et comparer les patients traités uniquement en soins ambulatoires et ceux traités à la fois en hospitalisation et en ambulatoire. Le test de *chi*-deux et une analyse de variance ont été exécutés lorsque cela était nécessaire. Les données qualitatives ont été analysés suivant l’approche cadre.
Résultats : Bien que les directives de soins nationales préconisent les soins ambulatoires pour le traitement primaire de la tuberculose en Chine, nous avons relevé des taux d’hospitalisation des patients tuberculeux allant de 39 % dans la circonscription de Yangzhong à 83 % dans celle de Dantu. Presque tous les tuberculeux soignés en consultations externes ont payé des tests de laboratoire, plus de 80 % des médicaments hépatoprotecteurs et 70 % des examens d’imagerie. Ces trois postes représentent les trois quarts des dépenses totales pour les soins en consultations externes. Pour les patients soignés uniquement en consultations externes, le coût total au terme du traitement de la tuberculose s’élevait en moyenne à 1135 yuans [environ 165 €/USD]. Pour les patients soignés à la fois en consultations externes et en hospitalisation, le coût total au terme du traitement de la tuberculose s’élevait en moyenne à 11 117 yuans [environ 1755 €/USD].
Conclusion : La politique de soins « gratuits » contre la tuberculose n’a pas été appliquée efficacement dans le

cadre du modèle intégré en Chine. Les patients ont dépensé des sommes considérables en prestations recommandations, examens et médicaments pour le traitement de la tuberculose.

Translated from English version into French by Suzanne Assenat, through

TRANSLATORS
WITHOUT BORDERS

Реализация политики «бесплатного» лечения туберкулеза (ТВ) в рамках интегрированной модели в провинции Цзянсу Китая: практика и стоимость в реальной жизни

Синьсинь Цзя[1], Цзяин Чэнь[1*], Сыюань Чжан[2], Бин Дай[3], Цянь Лун[4,5] и Шэньлан Тан[4,5]

Реферат

Предисловие: Китай ввел в 1990-х годах в рамках национальной программы контроля за ТВ политику «бесплатного» лечения туберкулеза (ТВ). Недавно было рекомендовано расширить интегрированную модель в виде части новой модели диагностики и лечения ТВ. В настоящей статье рассматривается вопрос, удалось ли специализированным ТВ-больницам обеспечить лечение ТВ в соответствии с национальными и местными директивами, и анализируются реальная практика и расходы, связанные с проведением лечения ТВ. В нем, также, рассматриваются причины, почему «бесплатное» лечение ТВ в Китае невозможно эффективно реализовать в рамках интегрированной модели.

Методы: Настоящее исследование было проведено в трех округах города Чжэньцзян провинции Цзянсу. Использованы смешанные методы, в которых сочетались пересмотр национальных и местных директив по контролю ТВ, проведение опросов ТВ-пациентов, сбор данных о стационарных и амбулаторных ТВ-пациентах больниц и проведение качественных интервью с участниками. Для количественного анализа данных по уездам и в целях сравнения данных от пациентов, получивших только амбулаторное лечение, с данными от пациентов, получивших как амбулаторное, так и стационарное лечение, использовалась описательная статистика. При необходимости, выполнялись анализ на основе критерия хи-квадрат и анализ дисперсии. Качественные данные анализировались на основе рамочного подхода.

Результаты: Несмотря на то, что в национальных директивах по лечению ТВ в качестве основы для лечения ТВ в Китае рекомендуется амбулаторное лечение, нами обнаружены высокие показатели госпитализации ТВ-пациентов в диапазоне от 39% в уезде Янчжун до 83% в уезде Даньту. Почти все амбулаторные пациенты платили за лабораторные анализы и 80% платили за лекарства для защиты печени, а около 70% платили за обследования с визуализацией. На эти три компонента приходятся три четверти от всех расходов амбулаторных больных. У пациентов, которые получили только амбулаторное лечение, общие затраты по завершении лечения ТВ составили в среднем 1135 юаней. У пациентов, получивших амбулаторное и стационарное лечение, общие расходы по завершении лечения ТВ составили 11117 юаней.

Заключение: Политика «бесплатного» лечения ТВ в рамках интегрированной модели не была эффективно реализована в Китае. Имелись существенные расходы на не рекомендованные услуги, обследования и лекарства для лечения ТВ.

Translated from English version into Russian by Alexander Somin, through

Cómo implementar una política de atención "gratuita" para la tuberculosis (TB) bajo el modelo integrado en Jiangsu, China: prácticas y costos en el mundo real

Xinxin Jia[1], Jiaying Chen[1*], Siyuan Zhang[2], Bing Dai[3], Qian Long[4,5] y Shenglan Tang[4,5]

Resumen

Antecedentes: En la década del 90 China introdujo una política de atención "gratuita" para la tuberculosis (TB) bajo el programa nacional de control de la TB. Recientemente, como parte del nuevo modelo de diagnóstico y tratamiento de la TB, se ha recomendado una ampliación del modelo integrado. Este artículo examina si los hospitales designados para la TB en los sitios seleccionados del proyecto ofrecieron o no asistencia para la TB de acuerdo a las pautas nacionales y locales, y analiza las prácticas actuales y los gastos asociados con el tratamiento de la TB. También explora los motivos por los cuales la asistencia "gratuita" para la TB en China no puede implementarse de manera efectiva bajo el modelo integrado.

Métodos: El estudio se llevó a cabo en tres condados de la ciudad de Zhenjiang, en la provincia de Jiangsu. Se utilizaron métodos combinados, que incluyeron la revisión de las pautas locales y nacionales para el control de la TB, la realización de encuestas a pacientes con TB, la recolección de registros hospitalarios de pacientes con TB, ambulatorios e internados y la realización de entrevistas a las partes involucradas. Se utilizaron estadísticas descriptivas para el análisis cuantitativo de la información en todos los condados y para comparar pacientes que recibieron solo asistencia ambulatoria y aquellos que recibieron tratamiento ambulatorio y estando hospitalizados. Se realizó la prueba de chi-cuadrado y un análisis de la varianza en aquellos casos que se consideró necesario. Se analizó la información mediante el marco cualitativo.

Resultados: Si bien las pautas nacionales para la TB recomiendan la atención ambulatoria como la base del tratamiento para la TB en China, encontramos un índice elevado de internación en hospitales para pacientes con TB que variaba entre el 39% en el condado de Yangzhong y el 83% en el condado de Dantu. Casi todos los pacientes ambulatorios con TB pagaron por sus análisis de laboratorio y más del 80% pagaron por los protectores hepáticos y cerca del 70% pagaron por las imágenes diagnósticas. Estos tres componentes explican tres-cuartos de los gastos ambulatorios totales. Para pacientes que recibieron solo atención ambulatoria, el gasto total al completar el tratamiento para la TB fue en promedio de 1.135 yuanes chinos. Para pacientes que recibieron atención ambulatoria y mientras estaban hospitalizados, el gasto total al completar el tratamiento para la TB fue en promedio de 11.117 yuanes chinos.

Conclusión: La política de atención "gratuita" para la TB bajo el modelo integrado no ha sido implementada de manera efectiva en China. Hubo un gasto significativo en servicios, exámenes y medicamentos que no son recomendados para el tratamiento de la TB.

Translated from English version into Spanish by Maria Alejandra Aguada, through
